# Conservative treatment, plate fixation, or prosthesis for proximal humeral fracture. A prospective randomized study

**DOI:** 10.1186/1471-2474-13-167

**Published:** 2012-09-07

**Authors:** Antti P Launonen, Vesa Lepola, Tapio Flinkkilä, Niko Strandberg, Johanna Ojanperä, Pekka Rissanen, Antti Malmivaara, Ville M Mattila, Petra Elo, Timo Viljakka, Minna Laitinen

**Affiliations:** 1 Department of Orthopaedics, Tampere University Hospital, Teiskontie 35, PL2000, Tampere 33521, Finland; 2 Oulu University Hospital, Kajaanintie 50, PL21, Oulu, 90029, Finland; 3 Department of Orthopaedics and Traumatology, Turku University Hospital, Kiinamyllynkatu 4-8, PL 52, Turku, 20521, Finland; 4 Department of Traumatology and Hand Surgery, Kuopio University Hospital, Puijonlaaksontie 2, PL1777, Kuopio, 70211, Finland; 5 University of Tampere, Tampere School of Public Health, Tampere, 33014, Finland; 6 National Institute for Health and Welfare, Mannerheimintie 166, PL 30, 00271, Helsinki, Finland; 7 Tampere University Hospital; Imaging Center, Biokatu 8, PL2000, Tampere, 33521, Finland; 8 Department of Hand Surgery, Tampere University Hospital, Teiskontie 35, PL2000, Tampere, 33521, Finland

**Keywords:** Proximal, Humerus, Fracture, Conservative, Operative, Locking plate, Prosthesis, Philos, Epoca, RCT

## Abstract

**Background:**

Proximal humerus fracture is the third most common fracture type after hip and distal radius fracture in elderly patients. A comprehensive study by Palvanen *et al*. demonstrated an increase in the annual fracture rate of 13.7% per year over the past 33 years. Should this trend continue, the fracture rate would triple over the next three decades. The increasing incidence of low-energy fractures raises questions about the optimal treatment in terms of functional outcome, pain, and rehabilitation time, as well as the economical impact. Despite the high incidence and costs of proximal humerus fractures, there is currently no valid scientific evidence for the best treatment method. Several publications, including a Cochrane review outline the need for high-quality, well-designed randomized controlled trials.

**Methods/Design:**

The study is a prospective, randomized, national multi-center trial. The hypothesis of the trial is that surgical treatment of displaced proximal humerus fractures achieves better functional outcome, pain relief, and patient satisfaction compared to conservative treatment. The trial is designed to compare conservative and surgical treatment of proximal humerus fractures in patients 60 years and older. The trial includes two strata. Stratum I compares surgical treatment with locking plates to conservative treatment for two-part fractures. Stratum II compares multi-fragmented fractures, including three- and four-part fractures. The aim of Stratum II is to compare conservative treatment, surgical treatment with the Philos locking plate, and hemiarthroplasty with an Epoca prosthesis. The primary outcome measure will be the Disabilities of the Arm, Shoulder and Hand (DASH) score and the secondary outcome measures will be the EuroQol-5D (EQ-5D) value, OSS, Constant-Murley Score, VAS, and 15D.

Recruiting time will be 3 years. The results will be analyzed after the 2-year follow-up period.

**Discussion:**

This publication presents a prospective, randomized, national multi-center trial. It gives details of patient flow, randomization, aftercare and also ways of analysis of the material and ways to present and publish the results.

**Trial registration:**

ClinicalTrials.gov identifier: NCT01246167

## Background

Proximal humerus fracture is the third most common fracture type after hip and distal radius fracture in elderly patients [1-3]. Proximal humerus fracture accounts for approximately 4% of all fractures [1-3]. Approximately 85% of the patients are treated conservatively and will regain shoulder function without surgery [4]. Most of these fractures are stable and minimally or non-displaced osteoporotic fractures and they commonly occur in women [4]. The mechanism of low-energy injury in elderly patients is usually falling from standing height. In Finland in 2002, the age-adjusted fracture incidence in persons 60 years and older was 105 per 100,000 person-years [5]. A comprehensive study by Palvanen *et al*. demonstrated an increase in the annual fracture rate of 13.7% per year over the past 33 years [5]. Should this trend continue, the fracture rate would triple over the next three decades.

The increasing incidence of low-energy fractures raises questions about the optimal treatment in terms of functional outcome, pain, and rehabilitation time, as well as the economical impact. Despite the high incidence and costs of proximal humerus fractures, there is currently no valid scientific evidence for the best treatment method. Several publications, including a Cochrane review outline the need for high-quality, well-designed randomized controlled trials. The challenge for the future is to determine which patients will benefit from surgery and to establish surgical techniques that produce optimal results for each fracture type. The aim of this randomized controlled trial is to evaluate whether the outcome in patients over 60-years of age with displaced two-, three-, and four-part fractures of the proximal humerus is improved by surgical intervention.

### Diagnosis and treatment

Diagnosis of proximal humerus fracture is based on clinical and radiologic findings and the mechanism of injury. A standard set of three radiographs from different views is generally obtained. The Neers’s or AO (Arbeitsgemeinschaft für Osteosynthesefragen) classification systems are widely used to define these complex fractures [6]. Although these systems have been used extensively for many decades, their reliability has been challenged. The AO system categorizes the fracture types into 27 fracture patterns, making its use labor- and time-intensive and complicated (Müller 1990). The Codman-Hertel binary fracture description system does not address the fracture pathomechanism (Hertel 2004). The Codman-Hertel system was improved by Resch by adding the pathomechanism of the fracture to the classification [7]. In all classification systems, however, the intra- and inter-observer agreement are graded as poor or, at best, moderate [8]. Due to poor intra- and inter-observer agreement of the Neer’s or AO classification systems, various radiographic protocols have been introduced to improve the diagnostic reliability of the classification. CT is often performed to facilitate treatment decisions.

Approximately 15% of patients with proximal humerus fracture are treated surgically [9]. Several fixation methods have been introduced, including Kirschner-wire fixation, screw fixation, plate fixation, intramedullary fixation, and prosthesis [10]. Currently, the locking plate system is the most frequently used method for fixation in two- and three-part fractures and a locking plate or prostheses is often used in displaced three- and four-part fractures in elderly patients [11]. With locking plates, the normal anatomy may be restored and the range of motion (ROM) is reported to recover up to 80% to 85% that of the healthy side [10,12]. The disadvantage of the locking plates includes a rather high complication rate of up to 49% [13]. A stable and usually pain-free shoulder is achieved with a prosthesis, but recovery of ROM is poor [10,12,14-17].

### Evaluation of treatment

Tools that are widely used for measuring the mobility and usability of the shoulder include the Constant-Murley score; Disabilities of the Arm, Shoulder and Hand (DASH) questionnaire; Oxford Shoulder Score (OSS); and Visual Analog Scale (VAS). In addition, the EuroQol-5D (EQ-5D) and 15D questionnaires survey the patient's general quality of life through different questions pertaining to various areas of life and are widely used in medical trials [18]. The outcomes are indexed and are comparable with reference populations as well as with the patient's own results in other stages of the treatment. Finnish versions of the EQ-5D and 15D have been validated [19,20].

### Previous studies

Although the literature on proximal humerus fractures is extensive, the majority of studies lack randomization, comparators, and independent evaluation, which makes it impossible to draw clinically meaningful conclusions [21]. Recent publications include three well done randomized, controlled trials. Olerud *et al.* carried out randomized controlled trials on three-part fractures, comparing nonsurgical treatment with angle-stable plates in elderly patients. The results indicated advantages in functional outcome and health-related quality of life favoring the locking plate, but the clinical significance remains unclear [22]. Fjalestad *et al.* studied displaced three and four-part fractures in patients over 60 years of age. They found no evidence that surgical treatment with an angle-stable device provided better results than conservative treatment [23]. Others have reported conflicting results. Olerud *et al.* studied displaced four-part fractures in elderly patients. They compared hemiarthroplasty and conservative treatment and found that arthroplasty provided a significant advantage in terms of quality of life. The main advantage was less pain although there was no difference in ROM [24].

In 2009, Hanson *et al.* published functional results of 160 patients treated conservatively. After a 12-month follow-up, the difference compared to the healthy side was 8.2 points measured with the Constant-Murley score. The difference in the DASH score was 10.2 points, which is below the minimal detectable change. Non-union risk was 7.0%, with smokers having a 5.5 times greater risk than nonsmokers [25].

The updated Cochrane meta-analysis published in 2010 was unable to provide guidelines for treating proximal humerus fractures due to a lack of solid evidence. Three- and four-part fractures in patients over 60 years of age are especially challenging as scientific consensus on their treatment has yet to be established [21].

## Methods/Design

The present randomized controlled trial is designed to compare conservative and surgical treatment of proximal humerus fractures. The trial includes two strata. Stratum I compares surgical treatment with locking plates to conservative treatment for two-part fractures. Stratum II compares multi-fragmented fractures, including three- and four-part fractures. The aim of Stratum II is to compare conservative treatment, surgical treatment with the Philos locking plate (Synthes®), and hemiarthroplasty with an Epoca prosthesis (Synthes®).

### Hypothesis

The study is a prospective, randomized, national multi-center trial. The hypothesis of the trial is that surgical treatment of displaced proximal humerus fractures achieves better functional outcome, pain relief, and patient satisfaction compared to conservative treatment in terms of ROM, and Constant-Murley, DASH, OSS, EQ-5D, 15D, and VAS scores. Subgroup analysis will be performed in an effort to obtain limit values for specific treatments of different age and fracture groups. In addition, we hypothesize that shoulder function will improve for up to 1 year from the time of fracture, and after 1 year significant improvement will be arrested [26]. The primary outcome measure will be the Disabilities of the Arm, Shoulder and Hand (DASH) score and the secondary outcome measures will be the EuroQol-5D (EQ-5D) value, OSS, Constant-Murley Score, VAS, and 15D.

### Objectives

The results of both strata will be analyzed and the results will be reported separately, as recommended by the CONSORT statement.

### Patients and methods

#### Inclusion criteria

• Low energy proximal humerus displaced (displacement more than 1 cm or 45 degrees) two-part fracture in which the fracture line emerges through the surgical (or anatomic) neck

• Low energy proximal humerus displaced (displacement more than 1 cm or 45 degrees) three- or four-part fracture

#### Exclusion criteria

• Refusal to participate in the study

• Under 60 years of age

• Not independent

• Dementia and/or institutionalized

• Does not understand written and spoken guidance in either Finnish or Swedish

• Pathologic fracture or a previous fracture of the same proximal humerus

• Alcoholism or drug addiction, e.g., in the emergency department, breathalyzer indicates blood alcohol concentration of more than 2 ‰

• Other injury to the same upper limb requiring surgery

• Major nerve injury (e.g., complete radial- or axillary nerve palsy)

• Rotator cuff tear arthropathy

• Open fracture

• Multi-trauma or -fractured patient

• Fracture dislocation or head-splitting fracture

• Non-displaced fracture

• Isolated fracture of the major or minor tubercle

• Gross displacement of the fracture fragments (no bony contact between fracture parts or the humerus shaft is in contact with the articular surface)

• Any medical condition that excludes surgical treatment

Patients with an x-ray verified proximal humerus fracture meeting the inclusion criteria will undergo a CT scan to assess the fracture classification. The scan area will include the entire scapula with the upper third of the humerus. Coronal, sagittal, and 3-dimensional volume reformats will be reconstructed. If the fracture meets the radiologic inclusion criteria, the patient will be invited to participate in the study. The patient will be informed of the study and will receive a written information sheet. When patients decide to participate in the study, they will be asked to fill-out a written informed consent form. The patients can withdraw from the study at any stage, on any grounds, and this will have no influence on the medical care given to the patients. If the patient is excluded from the study, information about age, sex, fracture type, reason for exclusion, medical condition, basic medication, and chosen treatment will be communicated to the research group using a case report form.

During hospitalization, the patient will be asked to fill out, with help if necessary, the EQ-5D, 15D, DASH, and OSS, and basic patient questionnaires with a VAS to determine their baseline characteristics. The patient's medical history, medication, and surgery will be recorded by a research nurse or researcher on a medical case report form. Potential primary complications will be recorded (e.g., nerve injury).

### Randomization

All patients will be randomized by a Tampere University Hospital research coordinator who will not attend the study. Patients with a two-part fracture will be randomized to either conservative or plate-fixation groups. Patients with multi-fragmented fractures will be randomized to conservative, plate fixation, or prosthesis groups. Both fracture types will be randomized using a random number matrix in block allocation fashion. The blocks will be age-dependent because, based on the literature, age and functional outcome are associated [27]. The treatment allocations from the matrix will be sealed in an envelope. After the patient's enrollment in the study has been confirmed, the research physician will contact the research coordinator, who will open the envelope and the randomized treatment will be carried out. The research coordinator will monitor the study flow. An independent monitoring committee has not been established.

### Surgical technique

The surgical procedures (plate fixation or hemiarthroplasty) are performed by shoulder-oriented orthopedic surgeons of the Tampere, Kuopio, Turku, and Oulu University hospitals. In this trial, we will use the Philos locking plate system (Synthes®, Solothurn, Switzerland) and an uncemented Epoca fracture prosthesis (Synthes®, Solothurn, Switzerland) with a hydroxyapatite coating. Additional cement fixation will be used if necessary. All patients randomized to surgical treatment will undergo the surgery within 2 weeks after the fracture. During the operation, the Neer’s classification and evidence of potential rotator cuff rupture will be recorded.

Patients will be placed in the beach-chair position. Plexus anesthesia will be used when possible. The deltopectoral, deltoid-split, or minimally invasive plate osteosynthesis approach will be used. Cuff tendons will be routinely inspected and sutures passed through each tendon to ease the handling of the fragments. For plating, the fragments will be preliminarily reduced with sutures and k-wires. The plate will be placed lateral from the biceps groove and 5 mm distal from the tip of the tuberculum majus. Six to eight locking screws will be placed in the head and three conventional or locking screws will be placed in the shaft.

The prosthesis height will be determined from the medial rim of the articular fragment. Anatomic retroversion will be determined from the shaft configuration. Reaming will be performed until the trial stem is stable. If needed, a minimal amount of cement will be used to stabilize the stem. Head size is determined from the articular fragment. The offset will be left to neutral. Before tightening the cables underneath the tuberculi, bone grafts from the head fragment will be placed against the stem. Sutures will be knotted to secure the cuff.

Drainage will be left in if necessary and the wound closed in layers. The shoulder will be immobilized with a collar-cuff in the operating theater.

### Conservative treatment

Patients randomized to non-operative treatment will be instructed with regard to joint mobilization by a physiotherapist during hospitalization. Patients will receive a written aftercare protocol with detailed pictures. A collar-cuff or a sling will be used for 3 weeks to relieve pain. During the first 3 weeks, pendulum exercises are allowed, and free joint mobilization and normal limb activation throughout treatment will be strongly supported. Active ROM exercises, as allowed by pain, will begin at 3 weeks. Physiotherapist contacts will be arranged to begin 3 and 6 weeks after surgery and all patients will have 5 physiotherapist contacts within the 3 first months.

### Postoperative aftercare

Patients operated with a plate will follow the same protocol as in conservative treatment. Patients treated with a prosthesis will wear a sling for 6 weeks. Two weeks postoperatively, they will begin pendulum movements. Free, active mobilization will be allowed at 6 weeks. Patients will be advised to mobilize their free joints from the beginning of the treatment and normal limb activation during aftercare will be supported. Contact with a hospital physiotherapist will begin after 3 and 6 weeks postoperatively and all patients will have 5 physiotherapist contacts within 3 months from the beginning of treatment. Patients will receive a detailed written aftercare protocol with instructional pictures and formal physiotherapy will be instructed before leaving the hospital.

### Follow-up

Follow-up will be carried out at the orthopedic outpatient clinic of the hospital where the patient was primarily treated. The patients will visit the outpatient clinic at 6 weeks and 3 months. Ultrasound examination of the fractured shoulder to assess possible rotator cuff injury will be performed at the 3-month visit by an experienced musculoskeletal radiologist.

Orthopedic outpatient clinic visits will be continued if necessary. In addition to these visits, in each center a blinded physiotherapist or sports physiologist will perform a research examination at 6 months, and 1, 2, 5, and 10 years from the beginning of the treatment. Patients are supposed to wear a shirt to blind the examiner. During these visits, radiographs will be taken of the treated shoulder, ROM and Constant-Murley scores of both shoulders will be obtained, and the patient will complete the EQ-5D, 15D, DASH, and OSS questionnaires.

**Figure 1:**
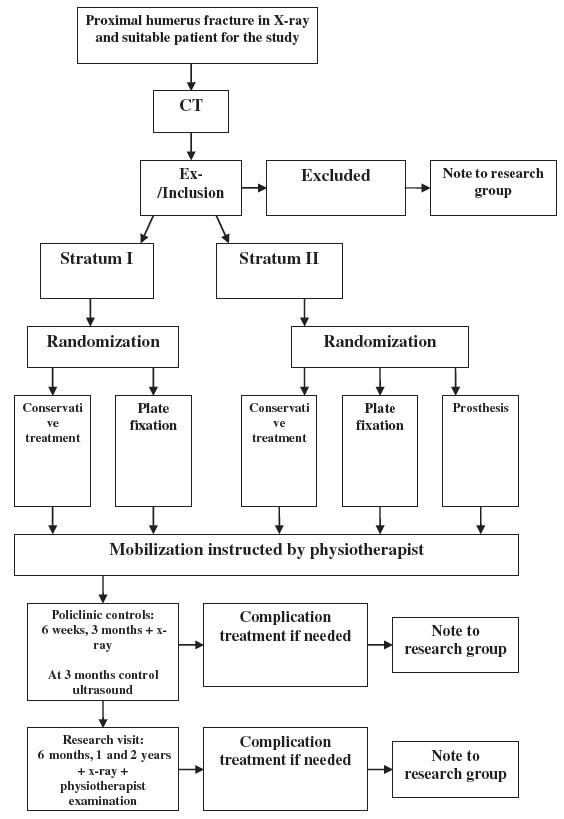
**Study flow. **Stratum I for 2 part fractures, Stratum II for 3 and 4 part fractures.

Should any adverse event demanding separate outpatient or inpatient care or surgery occur during the follow-up, an adverse event form will be completed within 24 hours of the execution of treatment. The information will be sent to the research coordinator. If the patient is not willing to continue in the study, or does not appear at appointments, or dies, a research discontinuation form will be completed. The study flow is outlined in Figure 1 and assessments and procedures are outlined in Table 1.

**Table 1:** Assessments and procedures of the trial

**Assessment**	**Preoperative**	**1. visit 6 weeks**	**2. visit 3 months**	**3. visit 6 months**	**4. visit 1 year**	**5.visit 2 years**
x-ray	X	X	X	X	X	X
CT	X					
Ultrasound			X			
Ex-/inclusion	X					
Medical history	X					
Consent	X					
Questionnaire	X		X	X	X	X
VAS-pain	X			X	X	X
EQ-5D	X			X	X	X
15D	X			X	X	X
OSS	X			X	X	X
DASH	X			X	X	X
Constant-Murley Score				X	X	X
Doctors visit		X	X			
Research visit				X	X	X

### Power analysis

In Stratum I, when assuming an effect size of a 10-point difference in the DASH score and a standard deviation of 15 points, the estimated sample size is 37 patients (delta = 10, sd = 15, alpha = 0.05, power = 0.8). Thus Stratum I requires 74 patients (2 comparison groups). In Stratum II, when assuming an effect size of a 10-point difference in the DASH score and a standard deviation of 15 points, the estimated sample size is 66 patients (ANOVA, alpha = 0.05, power = 0.8). The number of comparison groups is three. The total number of patients in Stratum II is 198. Thus, the total number of patients in the study will be 272. We will assume a 10% drop-out rate in both groups; therefore, the total patient number required will be 299 (81 + 218). In cases in which the patient changes to a different treatment group, they will be analyzed according to the intention-to-treat principle. SD value has been estimated after Gummerson *et al.* (2003). The article describes the minimal detectable change in DASH as 10 points with an SD of 13 with a mean score of 15 points [28].

### Statistical analysis

Differences between groups in continuous skewed main outcome variables will be analyzed by the Mann–Whitney U-test and t-test when variables are unskewed. Results are presented with 95% confidence intervals. Two-way-tables with the chi-square test will be used for dichotomous variables. Multivariate analysis will be conducted with regression analysis. In subgroup analysis the effect of age, sex, fracture group, smoking, and other diseases will be evaluated against the ROM, OSS, Constant-Murley, and overall quality of life after fracture.

### Analysis of the material

All radiographs and CT scans will be sent to the research center at Tampere University Hospital.

All information gathered will be stored in a study registry at Tampere University Hospital. The registry is protected with passwords, and will be deleted 2 years after the end of the study.

### Ethics

The trial protocol has been approved by the Ethics Committee of Pirkanmaa District Hospital. The study protocol and additional papers, including the consent form, patient information sheet, questionnaires, and case report form, have also been approved by the Ethics Committee (Approval number R10127). Permission to collect registry data and to combine it with the hospitalization data maintained by the NIHW will be requested from the NIHW and Social Insurance Institution.

### Time schedule

Recruiting time will be 3 years. The results will be analyzed after the 2-year follow-up period. The final report will be published by the end of the year 2017.

## Discussion

This publication presents a prospctive, randomized, national multi-center trial. It gives details of patient flow, randomization, aftercare and also ways of analysis of the material and ways to present and publish the results.

## Competing interests

The authors declare that they have no competing interests.

## Authors’ contributions

AL, ML, TF, JO, NS and TV are responsible for developing the trial. AL, VM, TF, PE, PR, AM, NS, TV, VL and ML drafted the protocol. AM will monitor and advise the methodologic aspects of the trial. PE is responsible for the radiologic aspects and PR is responsible for the economical aspect of the trial. VM performed the power calculations and determination of sufficient study group size and will perform the statistical analyses. All authors read and approved the final manuscript.

## Authors’ information

ML is the Principal Investigator of the study and she, as well as the Co-Principal Investigators in Oulu and Turku – TF and NS, are experienced in carrying out randomized controlled trials. TF, VL and NS are also nationally respected shoulder surgeons. JO is Co-Principal Investigator at Kuopio University Hospital. AL is the main researcher and is responsible for coordinating the medical aspects and the practical side of the study. TV is a nationally respected senior shoulder surgeon and will aid in planning the study and instructing junior colleagues. PE is an experienced musculoskeletal radiologist and will provide advice on radiologic issues and is the head of the radiologic board of the trial. PR is Professor of Public Health of University of Tampere and is responsible for the economical aspects of the study. VM is Associate Professor in the Department of Traumatology. AM is a researcher at the National Institute for Health and Welfare.

## Pre-publication history

The pre-publication history for this paper can be accessed here:

http://www.biomedcentral.com/1471-2474/13/167/prepub
